# A community based investigation of urban male partners’ understanding and participation in maternity care: A cross-sectional study in Bangladesh

**DOI:** 10.1371/journal.pone.0346873

**Published:** 2026-04-13

**Authors:** Quazi Maksudur Rahman, Md. Tajuddin Sikder

**Affiliations:** 1 Department of Public Health and Informatics, Jahangirnagar University, Savar, Dhaka, Bangladesh; 2 Department of Social Relations, East West University, Dhaka, Bangladesh; Public Library of Science, UNITED KINGDOM OF GREAT BRITAIN AND NORTHERN IRELAND

## Abstract

**Background:**

Male partners’ adequate understanding and engagement in maternity care plays a crucial role in enhancing women’s reproductive health. However, male involvement in maternal and child health care is still significantly below expectations, even though they perform key roles. Low knowledge levels appear to be a significant barrier to effective involvement.

**Objective:**

To examine the urban married men’s understanding and involvement in maternity care, as well as the factors associated with it.

**Methods:**

In this quantitative study, 422 data were collected through a structured questionnaire via face-to-face interviews between July and September 2022 among urban married males living in Dhaka City. The socio-demographic characteristics, level of understanding, males’ attitude and involvement status regarding maternity care were measured. The descriptive statistics along with logistic regression analysis were performed using STATA version 14.1.

**Results:**

A total of 422 urban married male were considered for this study, and the mean age of the study participants was 37.21 (SD ± 8.79). About 55.92% of the male participants had good understanding and 54% had positive attitude towards maternity care. Around 47% had good involvement in maternity care. Male who had good involvement in maternity care were more likely to have good understanding (AOR: 1.73; CI: 1.13 to 2.64; p = 0.01). In addition, urban male who had government jobs (AOR: 6.48; CI: 1.54 to 27.22; p = 0.01), positive attitude (AOR: 1.79; CI: 1.16 to 2.76; p = 0.01) and good understanding (AOR: 1.74; CI: 1.14 to 2.66; p = 0.01) were more likely to get involved in maternity care.

**Conclusion:**

Maternity care was well-understood and positively viewed by the most urban married males, but their level of involvement was far below the expected level. However, to strengthen safe motherhood initiatives in Bangladesh, it is important to properly address the understanding and participation of urban males in maternity care and other related factors.

## 1. Introduction

Access to competent obstetric care during pregnancy, labor, and the postpartum period is a crucial factor in the health of mothers and their babies [1]. The act of a male taking decision and care of his wife and children is known as male involvement in maternity care. The value of including males in initiatives that promote maternal, fetal, and child health is becoming more widely understood around the world [2]. Engaging males in maternal and newborn health can have significant health advantages for women and children in low- and middle-income countries according to emerging evidence and program experience, which can minimize maternal and newborn health complications [3]. Initiatives to encourage male participation in maternal and newborn health were recommended by the World Health Organization in 2015 to improve the care provided to women [4]. The lack of understanding by men about maternal health issues is a concern because it limits the ability of women to receive maternity care [5,6]. Positive involvement was hindered by low understanding levels, even after husbands’ interest and attempts to promote pregnant women’s health were generally high [7]. To ensure safe motherhood, men must be involved in promoting maternal health, which is a vital way to improve the health of mothers and their newborns [8–11]. Even though the Millennium Development Goals (MDGs) aim for maternal mortality reduction of up to three quarters by 2015, the maternal mortality remains unacceptably high. Involving men who are household decision-makers may help to lower this mortality. The 2015–2030 Sustainable Development Goals (SDGs) continue to address this [12]. Although there were several obstacles that prevented their involvement, men usually had strong internal motivation and attitudes towards their participation during pregnancy [11]. Bangladesh as a country of a patriarchy; still faces many difficulties in engaging male in family planning as well as reproductive health services. Culturally unique gender norms around maternity have an impact on men’s participation in maternity care. The deprivation conditions that prevent women from accessing resources that could provide them with alternative support during pregnancy exacerbate this dilemma. Furthermore, the absence of privacy in structures designed for maternal health care services prevents male partners from being present in the delivery room [13,26]. According to a previous study conducted in Bangladesh, two-thirds of husbands realized that women have unique rights linked to pregnancy and childbirth, and one-quarter addressed the name of three or more risk signs associated with pregnancy, childbirth, and postpartum. Furthermore, there were only thirty-eight percent of males who accompanied their wives when they needed care during emergencies related to pregnancy, labor, and the postpartum period [1], and ninety percent of men had never accessed services related to reproductive and maternal health [14]. Although males play important roles in maternity and child health (MCH) services, their involvement is still far below expectations [15,16], and it was highlighted in prior studies that increasing the utilization of institutional delivery can be achieved by participating in at least four high-quality ANC sessions and husbands were present with 47% of women who attended antenatal care (ANC) [1,25] as well as lack of understanding about pregnancy difficulties and risk indications has made men reluctant to participate in reproductive health services [17]. There are, however, relative lacks studies that have looked at married Bangladeshi men’s understanding of and involvement in maternity care in urban settings. To develop sound strategies and policies for ensuring and strengthening safe motherhood programs and initiatives in a developing country like Bangladesh to reduce maternal mortality ratio (MMR) and infant mortality rate (IMR), this study has been conducted to perceive more about the understanding and involvement status of urban married male in maternity care as well as the factors linked to it.

## 2. Methods and materials

### 2.1 Study design and setting

This study was quantitative along with cross-sectional in nature and conducted among urban married male partners residing in North and South City Corporation of Dhaka from July to September 2022. The inclusion criteria of the study population were married males whose age fall in the range between 15 to 69 [18], and whose wife’s age ranges fall between 15 to 49 years [14], (However, in Bangladesh, the legal age for marriage is 21 for boys and 18 for girls) [27] living in urban areas of Dhaka city, having at least one child, and show a willingness to participate in the study. People who did not meet the criteria were excluded from the study.

### 2.2 Sampling and sample size

This study followed convenience sampling technique due to the time and resource constraints along with the access barrier in respect of collecting eligible data from the male respondents living in urban areas of Dhaka city. However, this sampling technique was also used by several previous studies [11,14,19,20]. The city was divided into two regions, e.g. Dhaka North City Corporation and Dhaka South City Corporation from where data were collected by considering eligible married males. The desired sample size of the study was 422 using the following formula (Cochran):


$$n=\frac{z^{\text{2}}\times p(\text{1}-p)}{d^{\text{2}}}D$$

Here,

n = sample size

z = z-score associated with the chosen level of confidence interval (which is 1.96 at 95% confidence interval)

p = expected prevalence rate (50%)

d = standard error (5%)

D= non-response rate (10%)

### 2.3 Data collection tools and procedure

Following a critical analysis of relevant literature, a structured questionnaire and a consent form were developed. An overview of the study’s background, objectives, eligibility requirements, risks, as well as benefits of the study provided on the first page of the questionnaire, along with a declaration of confidentiality. Twenty respondents participated in a pilot study that was carried out by training research personnel to collect data, determine whether the questionnaires were understandable to the general public, and assess the methods’ capacity to collect data rapidly without imposing onerous conditions. After finishing the pilot study, we changed some questions’ wording to suit the respondents’ needs better. The purpose of the pilot study was also to evaluate the eligibility requirements of the respondents and ascertain whether the data demonstrated too much or insufficient range of variability. At every stage of the data collection and analysis process, we upheld the study’s credibility in order to ensure its validity. Credibility was further increased by making certain that each respondent understood the questionnaire and the ultimate objective of the study. After the pre-testing phase, data were collected via face-to-face interviews after explaining the significance of the study and obtaining consent. Are you willing to engage in this study? This was a question that had a “Yes or No” response option. The complete questionnaire was made available to the respondents, and the interviews were conducted if the responses were positive. This study maintained ethical standards to the highest possible extent and the informed consent obtained from each male participant. All procedures were conducted following the Helsinki Declaration of 1964*.* This research was approved by Biosafety, Biosecurity, and Ethical Review Board of Jahangirnagar University, Savar, Dhaka-1342, Bangladesh [BBEC, JU/ M 2022/2(5)]. To protect the confidentiality of the data, all responses were made anonymous. After being informed about the study’s purpose, all male participants gave their written informed consent to participate. A total of 422 data was considered for final analysis by following the sample size estimation. There were no incentives provided to study participants for their participation. Confidentiality and data privacy were effectively protected at every turn.

### 2.4 Measures

The questionnaire comprised of (i) socio-demographic information, (ii) understanding scenario regarding maternity care, (iii) attitudes toward maternity care, and (iv) the level of involvement in maternity care.

#### 2.4.1 Socio-demographic information.

The socio-demographic information included respondents’ age structure (18-25, 26-33, 34-41, 42 or more), educational background (Up to SSC, HSC, diploma, undergraduate, graduate, above graduate), occupation (government job, private job, business, student and others), marital status (family or love arranged marriage), family size (nuclear or combined), the number of children (1, 2–4, 5–7, ≥ 8), monthly family income (< 20,000 BDT, 20,000–35,000 BDT, 35,001–50,000 BDT, and > 50,000 BDT), religious status (Muslim, non-Muslim) and current living locations etc.

#### 2.4.2 Understanding regarding maternity care.

It included 7 questions regarding understanding towards antenatal care with ‘Yes’ or ‘No’ response options based on four visits of ANC (1st: 8–12 weeks, second: between 24 and 26 weeks, third: at 32 weeks and fourth: at 36–38 weeks), screen of high-risk cases, provision of ongoing primary preventive healthcare, education of mothers about the physiology of pregnancy and labor to remove fear, discussion with the couple considering place, time and mode of delivery provisionally and care of the newborn, motivation towards couple about the need for family planning, and treatment or prevention of any earliest complications. Understanding towards intranatal care comprised of 4 questions including aseptic approach (cleaning hands, surface, blade, cord, and tie), delivery with minimum injury to the infant and mother, readiness to deal with complications, and care for the baby at the time of delivery with ‘Yes’ or ‘No’ response options. Apart from that, understanding towards postnatal care consisted of 6 questions with ‘Yes’ or ‘No’ response options including promotion of mother’s and baby’s physical well-being, usual physiological changes, help mother to establish a satisfactory feeding routine and relationship with the baby, teach care of the baby and strengthen women’s own confidence, optimal breastfeeding technique, and immunization of the infant etc. The ‘Yes’ and ‘No’ responses were scored one and zero, respectively. Understanding status was considered as good (greater than or equal to the mean score) and poor (less than the mean score) following the previous study [10,21].

#### 2.4.3 Attitude towards maternity care.

The attitude section included nine statements regarding maternity care by using 5-point Likert Scales comprised of ‘Strongly Agree’, ‘Agree’, ‘No Opinion’, ‘Disagree’, and ‘Strongly Disagree’ ranges from 5 to 1 respectively, such as men should encourage their wives to go for antenatal care, accompany their wives for antenatal care, encourage family planning, decide places of delivery, be present in the labor room, support their wives to attend postnatal visits, assist in house chores for the well-being of their wives, support exclusive breast-feeding and encourage their complete child immunization etc. The overall score was measured, and urban married males who scored below the mean were regarded as having a negative attitude, while those with scores equal to or above the respective mean were regarded as having a positive attitude towards maternity care by considering the following studies [10,22].

#### 2.4.4 Involvement or participation in maternity care.

The involvement of urban married male in antenatal care consisted of 8 questions with ‘Yes’ or ‘No’ response options, including staying with their wife at the time of pregnancy, involving in planned pregnancy, putting money aside for an emergency, involving in making transport arrangements, involving in a joint plan in an emergency, discussing maternal health issues with their wife’s healthcare providers during pregnancy, accompanying their wife for antenatal visits to the healthcare facilities, providing any support during pregnancy. As well as male partner’s involvement in intranatal care comprised of 5 questions with ‘Yes’ or ‘No’ response options including accompanying their wife at the time of labor or delivery, making a prior joint plan for labor or delivery, accompanying their wife to the healthcare facilities during labor or delivery, making discussion about maternal health issues with their wife’s healthcare providers during labor or delivery, providing any support during labor or delivery. In respect of postnatal care, another 5 questions were included with ‘Yes’ or ‘No’ response options including staying with their wife after delivery, making a joint plan during the post-delivery period, accompanying your wife for postnatal visits to the healthcare facilities, making discussion about maternal health issues with their wife’s healthcare providers after delivery, and providing any support after delivery. The ‘Yes’ and ‘No’ responses were scored using binary digits like one and zero, respectively. The male respondents who scored below the mean were regarded as having poor involvement, while those with scores equal to or above the respective mean were regarded as having good involvement by following the prior studies [10,21].

### 2.5 Data management and analysis

Data were cleaned, sorted, and coded using Microsoft Excel 2010. The descriptive statistics and logistic regression analysis were performed using STATA version 14.1 (StataCorp LP, College Station, TX, USA). The odds ratio (OR) with a 95% confidence interval (CI) was used to present the outcome of the regression analysis, and the adjusted odds ratio (AOR) was also calculated, taking into account all the study variables. If the p-values were 0.05 or lower, the variables’ association was considered to be deemed significant.

## 3. Results

### 3.1 Socio-demographic characteristics

The mean age of the study participants was 37.21 (SD ± 8.79), and 05.21% of the male participants were in the age group of eighteen to twenty-five. On the contrary, the majority of the urban male respondents, about 35.78% belonged to the age group of twenty-six to thirty-three. Around 42.18% were graduates when considering their educational background. Regarding their occupation, around 39% male involved in business and 35.31%, 20.38%, and 3.32% urban male were private job holders, government job holders, and students, respectively. In this study, about 66.11% and 33.89% got married by considering arranged and love marriage. Most of the male participants, about 66.59% belonged to the nuclear family. About 35.55% and 11.85% had monthly family income in the range of 35,001–50,000 BDT and < 20,000 BDT respectively [1 USD = 122.94 BDT]. In this study, the majority of the male respondents, around 84.83% came from respected Muslim families. (Table 1).

**Table 1:** Socio-demographic information of urban married male (N = 422).

Variables	Category	Frequency (N)	Percentage (%)
Age	18-25	22	05.21
	26-33	151	35.78
	34-41	127	30.09
	42 or more	122	28.91
	Mean age (37.21, SD ± 8.79)		
Education	Up to SSC	70	16.59
	HSC	66	15.64
	Diploma	05	01.18
	Undergraduate	56	13.27
	Graduate	178	42.18
	Above Graduate	47	11.14
Occupation	Government job	86	20.38
	Private job	149	35.31
	Business	165	39.10
	Student	14	03.32
	Others	08	01.90
Marital status	Family arranged marriage	279	66.11
	Love marriage	143	33.89
Family size	Nuclear	281	66.59
	Combined	141	33.41
No. of Children	1	168	39.81
	2-4	232	54.98
	5-7	20	04.74
	≥ 8	02	00.47
Monthly Family Income (BDT)	< 20,000	50	11.85
	20,000-35,000	131	31.04
	35,001-50,000	150	35.55
	> 50,000	91	21.56
Religion	Muslim	358	84.83
	Non-Muslim	64	15.17
Location	Dhaka North City Corporation	211	50.00
	Dhaka South City Corporation	211	50.00

### 3.2 Urban married males’ understanding towards maternity care

**Fig 1:**
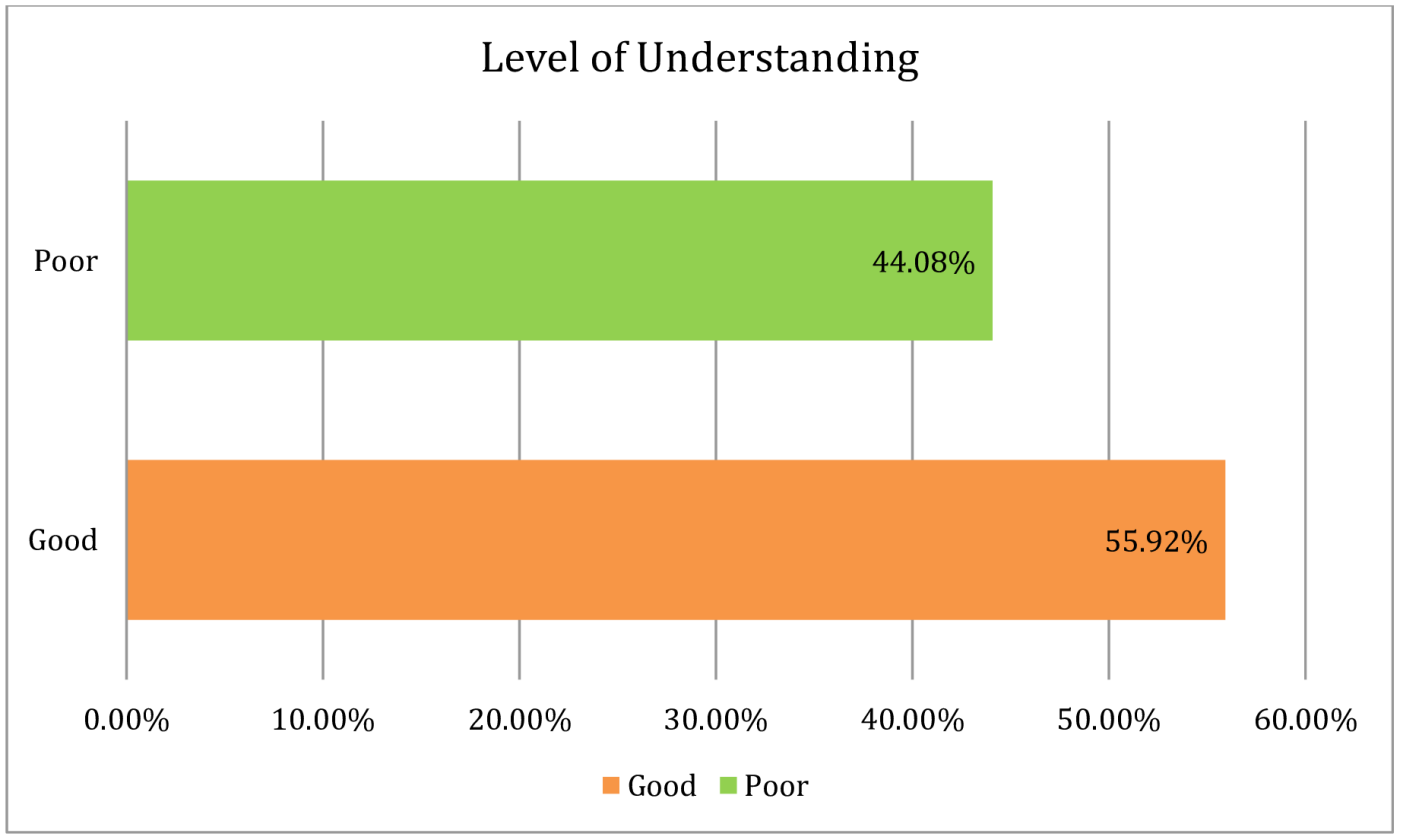
Urban married male partners’ understanding towards maternity care. According to this research, around 55.92% of the male participants had good understanding, and 44.08% of the male participants had poor understanding level of maternity care in respect of urban setting (Dhaka) in Bangladesh.

This study found that about 55.92% of the male participants had a good understanding of maternity care in respect of urban settings in Bangladesh (Fig 1). Regarding antenatal care (ANC), it disclosed that 49.29% (n = 208) urban male had a good level of understanding, and 50.71% (n = 214) had poor level of understanding. In the phase of antenatal care, the urban male had positive view of understanding regarding ANC like comprehending four antenatal visits (1st: 8–12 weeks, second: between 24 and 26 weeks, third: at 32 weeks and fourth: at 36–38 weeks (52.37%), screening high-risk cases (63.27%), providing ongoing primary preventive healthcare (82.46%) along with other services (Table 2). When considering the phase of intranatal care (INC), it was revealed that majority of the study respondents 60.90% (n = 257) had a good level of understanding, and about 39.10% had poor understanding. In the period of intranatal care (INC), the study respondents had a positive views of understanding towards intranatal care services comprising aseptic approach like trying to clean the surface, blade, cord, and tie (62.32%), ensuring delivery with minimum injury to infant and mother (74.88%), involving readiness to deal with complications (66.35%), and about 69.67% provided care of the baby at the time of delivery (Table 2). When considering the phase of postnatal care, it found that the majority of the male respondents 52.13% (n = 220) had a good level of understanding, and about 47.87% had poor knowledge or understanding. In respect of postnatal care, the positive response highlighted in their understanding regarding PNC like promoting the physical well-being of the mother and baby 80.81% (n = 341), ensuring physiological changes are normally occurring 75.59% (n = 319), establishing a satisfactory feeding routine and relationship with baby 51.90% (n = 219), teaching care of the baby and strengthening women’s own confidence 75.59% (n = 319) along with other services (Table 2).

**Table 2:** Urban married male partners’ understanding towards maternity care.

Maternity Care	Category		Understanding Status n (%)	
**Antenatal Care (ANC)**	**Yes (%)**	**No (%)**	**Good**	**Poor**
Comprising 4 visits (1st: 8–12 weeks, 2nd: between 24 and 26 weeks, 3rd: 32 weeks and 4th: at 36–38 weeks)	221(52.37)	201(47.63)	208(49.29)	214(50.71)
Screening of high-risk cases	267(63.27)	155(36.73)		
Providing ongoing primary preventive healthcare	348(82.46)	74(17.54)		
Educating mothers about the physiology of pregnancy and labor to remove fear.	322(76.30)	100(23.70)		
Discussing the couple about the place, time and mode of delivery, provisionally and care of newborn	278(65.88)	144(34.12)		
Motivating the couple about the need for family planning	234(55.45)	188(44.55)		
Treating or preventing any earliest complications	253(59.95)	169(40.05)		
**Intranatal Care (INC)**				
Comprising aseptic approach (Cleaning hands, surface, blade, cord, and tie)	263(62.32)	159(37.68)	257(60.90)	165(39.10)
Ensuring delivery with minimum injury to infant and mother	316(74.88)	106(25.12)		
Involving readiness to deal with complications	280(66.35)	142(33.65)		
Providing care of baby at the time of delivery	294(69.67)	128(30.33)		
**Postnatal Care (PNC)**				
Promoting the physical well-being of the mother and baby	341(80.81)	81(19.19)	220(52.13)	202(47.87)
Ensuring physiological changes are occurring normally	319(75.59)	103(24.41)		
Helping mother to establish a satisfactory feeding routine and relationship with baby	219(51.90)	203(48.10)		
Teaching care of the baby and strengthening women’s own confidence	319(75.59)	103(24.41)		
Supporting optimal breast-feeding technique?	345(81.75)	77(18.25)		
Ensuring immunization of the infant	347(82.23)	75(17.77)		

The bivariate logistic regression revealed that males who were undergraduate (OR: 2.11; CI: 1.01 to 4.38; p = 0.05) were more likely to have a good understanding of maternity care, and both the bivariate and multivariate logistic regression model showed that males who had good involvement status (OR: 1.77; CI: 1.20 to 2.62; p < 0.05) and (AOR: 1.73; CI: 1.13 to 2.64; p < 0.05) respectively were more likely to have a good understanding of maternity care (Table 3).

**Table 3:** Logistic regression model of factors associated with male partners’ understanding towards maternity care.

Variables	Understanding Status n (%)		COR (95% CI)	P value	AOR (95% CI)	P value
	Good	Poor				
Age						
18-25	13(5.51)	09 (04.84)	Ref.		Ref.	
26-33	88(37.29)	63(33.87)	0.97(0.39 to 2.40)	0.94	0.89(0.31 to 2.55)	0.83
34-41	76(32.20)	51(27.42)	1.03(0.41 to 2.59)	0.95	0.89(0.28 to 2.80)	0.84
42 or more	59(25.00)	63(33.87)	0.65(0.26 to 1.63)	0.36	0.44(0.13 to 1.49)	0.19
Education						
Up to SSC	35(14.83)	35(18.82)	Ref.		Ref.	
HSC	39(16.53)	27(14.52)	1.44(0.73 to 2.85)	0.29	1.48(0.68 to 3.24)	0.33
Diploma	03(1.27)	02(1.08)	1.50(0.24 to 9.54)	0.67	1.57(0.22 to 11.46)	0.65
Undergraduate	38(16.10)	18(09.68)	2.11(1.01 to 4.38)	0.05	2.01(0.83 to 4.88)	0.12
Graduate	96(40.68)	82(44.09)	1.17(0.67 to 2.04)	0.58	1.26(0.59 to 2.65)	0.55
Above graduate	25(10.59)	22(11.83)	1.14(0.54 to 2.38)	0.74	1.10(0.41 to 2.93)	0.85
Occupation						
Student	08(03.39)	06(03.23)	Ref.		Ref.	
Government job	46(19.49)	40(21.51)	0.86(0.28 to 2.70)	0.80	0.95(0.24 to 3.67)	0.94
Private job	83(35.17)	66(35.48)	0.94(0.31 to 2.85)	0.92	1.16(0.32 to 4.14)	0.82
Business	97(41.10)	68(36.56)	1.07(0.36 to 3.22)	0.90	1.13(0.31 to 4.09)	0.85
Others	02(00.85)	06(03.23)	0.25(0.04 to 1.70)	0.16	0.43(0.05 to 3.56)	0.44
Marital status						
Family marriage	162(68.64)	117(62.90)	Ref.		Ref.	
Love marriage	74(31.36)	69(37.10)	0.77(0.52 to 1.16)	0.22	0.72(0.46 to 1.11)	0.14
Family size						
Nuclear	154(65.25)	127(68.28)	Ref.		Ref.	
Combined	82(34.75)	59(31.72)	1.15(0.76 to 1.73)	0.51	1.08(0.70 to 1.69)	0.72
Number of children						
1	92(38.98)	76(40.86)	Ref.		Ref.	
2-4	132(55.93)	100(53.76)	1.09(0.73 to 1.63)	0.67	1.33(0.81 to 2.19)	0.26
5-7	12(05.08)	08(04.30)	1.24(0.48 to 3.19)	0.66	1.55(0.52 to 4.66)	0.43
≥ 8	00(00.00)	02(01.08)	1.00	–	1.00	–
Monthly family income (BDT)						
< 20,000	28(11.86)	22(11.83)	Ref.		Ref.	
20,000-35,000	72(30.51)	59(31.72)	0.96(0.50 to 1.85)	0.90	0.69(0.32 to 1.51)	0.35
35,001-50,000	81(34.32)	69(37.10)	0.92(0.48 to 1.76)	0.81	0.81(0.34 to 1.90)	0.63
> 50,000	55(23.31)	36(19.35)	1.20(0.60 to 2.41)	0.61	1.31(0.51 to 3.41)	0.58
Religion						
Muslim	202(85.59)	156(83.87)	Ref.		Ref.	
Non-Muslim	34(14.41)	30(16.13)	0.88(0.51 to 1.49)	0.63	0.78(0.43 to 1.40)	0.40
Attitude						
Negative	107(45.34)	89(47.85)	Ref.		Ref.	
Positive	129(54.66)	97(52.15)	1.11(0.75 to 1.63)	0.61	1.05(0.69 to 1.61)	0.83
Involvement status						
Poor	110(46.61)	113(60.75)	Ref.		Ref.	
Good	126(53.39)	73(39.25)	1.77(1.20 to 2.62)	0.004	1.73(1.13 to 2.64)	0.01

### 3.3 Urban married males’ attitude towards maternity care

In respect of urban male partners’ attitude, the findings revealed that about 54% of the study participants had a positive attitude, which was comparatively higher than those participants holding negative attitudes (46%) towards maternity care (Table 4). This study found that 42.89% respondents agreed that men should encourage their wives to go for antenatal care, and among the study participants, 43.13%, 41.47%, 47.16%, and 34.36% also agreed that men should accompany their wives, encourage family planning, decide places of delivery, and be present in the labor room respectively, whereas 0.47%, 0.24%, 0.71%, and 2.13% urban married male strongly disagreed regarding the above mentioned statement respectively (Table 4). In addition, about 40.05%, 40.52%, 48.82%, and 48.34% respondents strongly agreed with the statements that man should support their wives to attend postnatal visits, assist in house chores for the well-being of their wives, encourage their child complete immunization, and support exclusive breastfeeding respectively regarding maternity care (Table 4).

**Table 4:** Urban married male partners’ attitude towards maternity care.

	Level of attitude n (%)				
Activity	Strongly Agree	Agree	No-opinion	Disagree	Strongly Disagree
Men should encourage their wives to go for antenatal care	164(38.86)	181(42.89)	68(16.11)	09(02.13)	00(0.00)
Men should accompany their wives for antenatal care	150(35.55)	182(43.13)	80(18.96)	08(01.90)	02(00.47)
Men should encourage family planning	142(33.65)	175(41.47)	93(22.04)	11(02.61)	01(00.24)
Men should decide on places of delivery	116(27.49)	199(47.16)	92(21.80)	12(02.84)	03(00.71)
Men should be present in the labor room	133(31.52)	145(34.36)	103(24.41)	32(07.58)	09(02.13)
Men should support their wives in attending postnatal visits	169(40.05)	160(37.91)	82(19.43)	11(02.61)	00(0.00)
Men should assist in house chores for the well-being of their wives	171(40.52)	156(36.97)	84(19.91)	11(02.61)	00(0.00)
Men should encourage their complete child immunization	206(48.82)	171(40.52)	40(09.48)	05(01.18)	00(0.00)
Men should support exclusive breastfeeding	204(48.34)	173(41.00)	39(09.24)	05(01.18)	01(00.24)
Positive attitude: 226(54%) Negative attitude: 196 (46%)					

### 3.4 Urban married males’ involvement in maternity care

**Fig 2:**
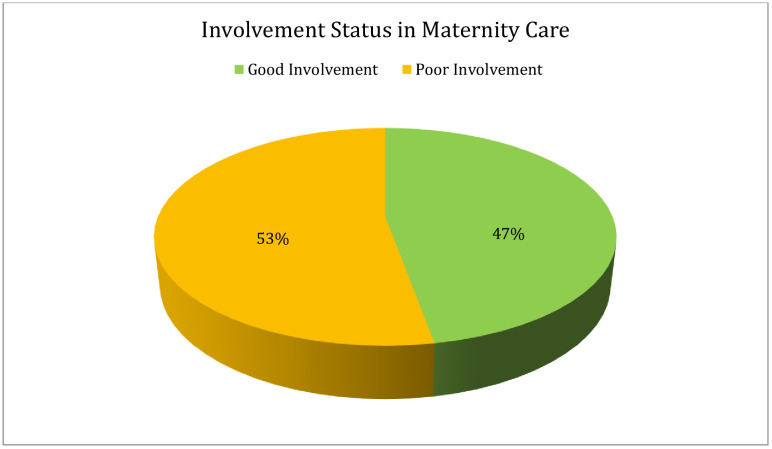
Urban married male partners’ involvement status in respect of maternity care. Among the study participants, about 47% of married men living in Dhaka city were actively (good) involved in maternity care, while 53% were not (poor) involved considering the context of Bangladesh.

**Fig 3:**
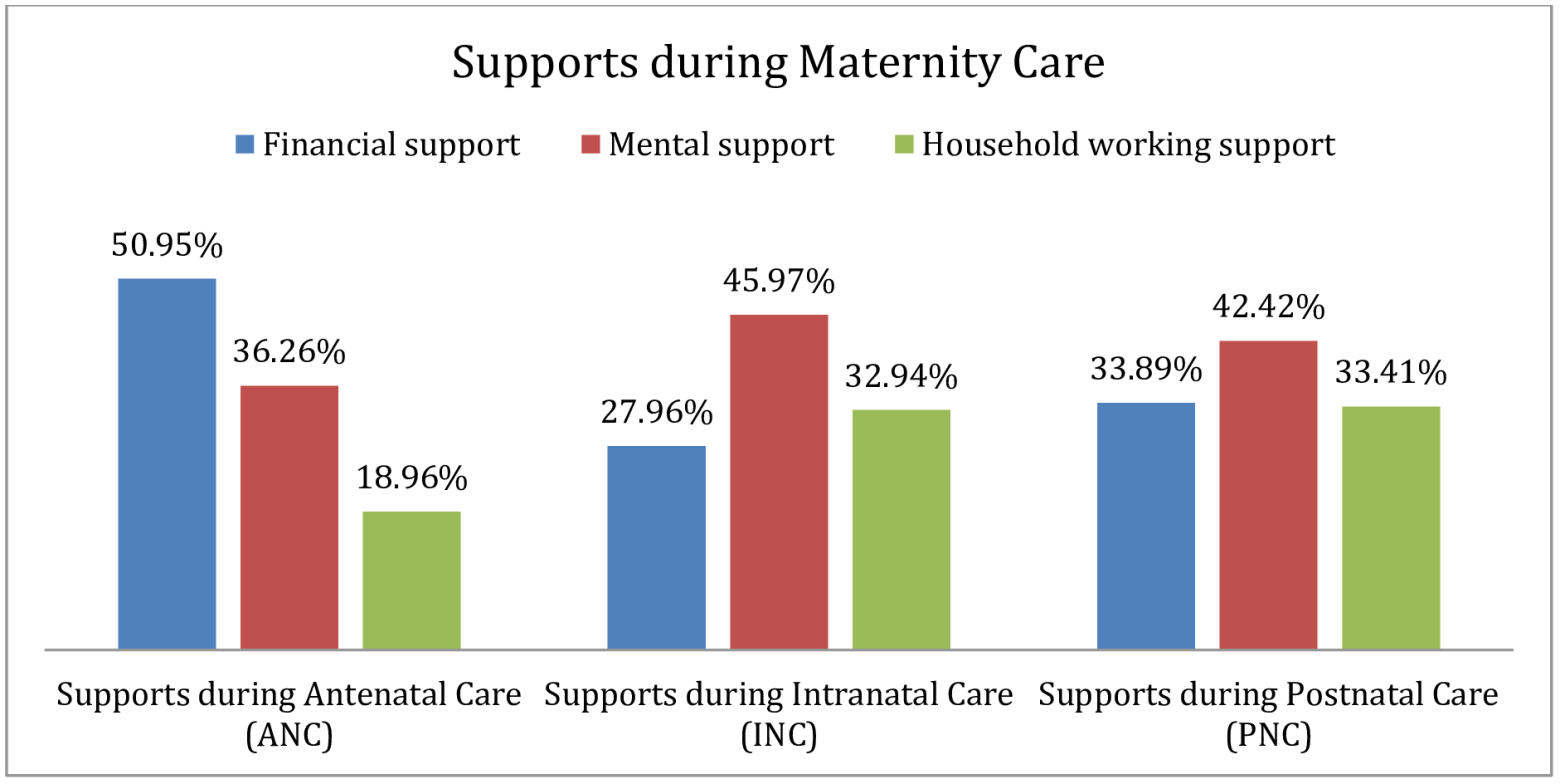
Urban married male partners’ supports in maternity care. The noticeable number of study participants during intranatal care (45.97%) gave mental support, and 33.41% during postnatal care, and 50.95% during antenatal care provided household working support and financial support, respectively.

Around 47% of urban married males had good involvement, and 53% had a poor engagement in maternity care in Bangladesh (Fig 2). In this study, about 43.84% (n = 185) of the study respondents accompanied with their wives at the time of pregnancy. Among the study respondents, 73.22% (n = 309), 81.28% (n = 343), 75.83% (n = 320), and 62.09% (n = 262) male involved in planned pregnancy, put money aside for emergency, made transport arrangements as well as involved in joint plans in emergency situations respectively during antenatal care (Table 5). In addition, the majority of them (50.95%) gave financial support besides mental support (36.26%) along with household working support (18.96%) during ANC (Fig 3). In respect of intranatal care, 50% (n = 211) of urban male accompanied their wife at the time of labor, and the same portion of the study respondents were unable to get involved during the intranatal phase of maternity care in Bangladesh (Table 5). During intranatal care, most of male (45.97%) gave mental support, and 33.41% (PNC), and 50.95% (ANC) provided household working support and financial support, respectively (Fig 3). About 43.13% (n = 182) stayed with their wife during postnatal care after delivery. The result also revealed that 56.87% (n = 240), 36.73% (n = 155), 36.02%(n = 152), 32.46% (n = 137), and 01.18% (n = 05) male participants not involved in respect of staying with wife after delivery, making joint plan during the post-delivery period, accompanying wife for postnatal visits to the healthcare facilities, making discussion on maternal health issues with their wife’s healthcare providers after delivery, and providing any supports after delivery (Table 5). Among the study respondents, the majority of them (42.42%) provided mental support along with 33.41%, 33.89% gave household and financial support, respectively, in the phase of postnatal care (Fig 3).

**Table 5:** Urban married male partners’ involvement in maternity care.

Maternity Care	Category	Frequency (n)	Percentage (%)
**Antenatal Care (ANC)**			
Did you give accompany to your wife at the time of pregnancy?	Yes	185	43.84
	No	237	56.16
Did you get involved in planned pregnancy?	Yes	309	73.22
	No	113	26.78
Did you put money aside for an emergency?	Yes	343	81.28
	No	79	18.72
Did you involve in making transport arrangements?	Yes	320	75.83
	No	102	24.17
Did you involve in a joint plan in an emergency situation?	Yes	262	62.09
	No	160	37.91
Did you discuss maternal health issues with your wife’s healthcare providers during pregnancy?	Yes	233	55.21
	No	189	44.79
Did you give accompany to your wife for antenatal visits to the healthcare facilities?	Yes	214	50.71
	No	208	49.29
Did you provide any support during pregnancy?	Yes	410	97.16
	No	12	02.84
**Intranatal Care (INC)**			
Did you give accompany to your wife at the time of labor/delivery?	Yes	211	50.00
	No	211	50.00
Did you make a prior joint plan for labor/delivery?	Yes	298	70.62
	No	124	29.38
Did you give accompany to your wife to the healthcare facilities during labor/delivery?	Yes	309	73.22
	No	113	26.78
Did you discuss maternal health issues with your wife’s healthcare providers during labor/delivery?	Yes	318	75.36
	No	104	24.64
Did you provide any support during delivery?	Yes	405	95.97
	No	17	04.03
**Postnatal Care (PNC)**			
Did you give accompany to your wife after delivery?	Yes	182	43.13
	No	240	56.87
Did you make a joint plan during the post-delivery period?	Yes	267	63.27
	No	155	36.73
Did you give accompany to your wife for postnatal visits to the healthcare facilities?	Yes	270	63.98
	No	152	36.02
Did you discuss maternal health issues with your wife’s healthcare providers after delivery?	Yes	285	67.54
	No	137	32.46
Did you provide any support after delivery?	Yes	417	98.82
	No	05	01.18

The bivariate logistic regression analysis showed that urban married males whose age range was forty-two or more (OR: 0.37; CI: 0.14 to 0.95; p < 0.05) were less likely to get involved in maternity care, whereas urban married male partners who had monthly family income around 20,000 BDT to 35,000 BDT (OR: 2.05; CI: 1.05 to 4.00; p < 0.05), positive attitude (OR: 1.89; CI: 1.28 to 2.78; p = 0.001), good level of understanding (OR: 1.77; CI: 1.20 to 2.62; p < 0.05) were more likely to get involved in maternity care in Bangladesh (Table 6). The multivariate logistic regression analysis further showed that the urban male aged 42 or more (AOR: 0.26; CI: 0.07 to 0.97; p < 0.05) were less likely to get engaged in maternity care. The urban married males who were undergraduate (AOR: 2.73; CI: 1.11 to 6.69; p < 0.05), had government jobs (AOR: 6.48; CI: 1.54 to 27.22; p < 0.05), positive attitude towards maternity care (AOR: 1.79; CI: 1.16 to 2.76; p < 0.05) and good level of understanding (AOR: 1.74; CI: 1.14 to 2.66; p < 0.05) were more likely to get involved in maternity care (Table 6).

**Table 6:** Logistic regression model of factors associated with urban married male partners’ involvement in maternity care.

Variables	Involvement Status n (%)		COR (95% CI)	P value	AOR (95% CI)	P value
	Good	Poor				
Age						
18-25	14(07.04)	08(03.59)	Ref.		Ref.	
26-33	72(36.18)	79(35.43)	0.52(0.21 to 1.31)	0.17	0.29(0.09 to 0.96)	0.04
34-41	65(32.66)	62(27.80)	0.60(0.24 to 1.53)	0.28	0.31(0.09 to 1.09)	0.07
42 or more	48(24.12)	74(33.18)	0.37(0.14 to 0.95)	0.04	0.26(0.07 to 0.97)	0.04
Education						
Up to SSC	26(13.07)	44(19.73)	Ref.		Ref.	
HSC	32(16.08)	34(15.25)	1.59(0.80 to 3.16)	0.18	1.65(0.74 to 3.71)	0.22
Diploma	01(00.50)	04(01.79)	0.42(0.04 to 3.99)	0.45	0.45(0.04 to 5.13)	0.52
Undergraduate	30(15.08)	26(11.66)	1.95(0.96 to 3.99)	0.07	2.73(1.11 to 6.69)	0.03
Graduate	89(44.72)	89(39.91)	1.69(0.96 to 2.98)	0.07	1.94(0.89 to 4.22)	0.10
Above graduate	21(10.55)	26(11.66)	1.37(0.64 to 2.90)	0.42	1.83(0.66 to 5.07)	0.25
Occupation						
Student	05(02.51)	09(04.04)	Ref.		Ref.	
Government job	50(25.13)	36(16.14)	2.50(0.77 to 8.09)	0.12	6.48(1.54 to 27.22)	0.01
Private job	63(31.66)	86(38.57)	1.32(0.42 to 4.13)	0.64	2.55(0.65 to 9.99)	0.18
Business	80(40.20)	85(38.12)	1.69(0.54 to 5.27)	0.36	3.96(1.01 to 15.53)	0.05
Others	01(00.50)	07(03.14)	0.26(0.02 to 2.73)	0.26	0.58(0.04 to 8.00)	0.68
Marital status						
Family marriage	129(64.82)	150(67.26)	Ref.		Ref.	
Love marriage	70(35.18)	73(32.74)	1.12(0.74 to 1.67)	0.60	1.22(0.77 to 1.91)	0.40
Family size						
Nuclear	131(65.83)	150(67.26)	Ref.		Ref.	
Combined	68(34.17)	73(32.74)	1.07(0.71 to 1.60)	0.76	1.12(0.71 to 1.75)	0.63
Number of children						
1	89(44.72)	79(35.43)	Ref.		Ref.	
2-4	103(51.76)	129(57.85)	0.71(0.48 to 1.06)	0.09	0.69(0.42 to 1.15)	0.16
5-7	07(03.52)	13(05.83)	0.48(0.18 to 1.26)	0.14	0.62(0.20 to 1.96)	0.42
≥ 8	00(00.00)	02(00.90)	1.00	–	1.00	–
Monthly family income (BDT)						
< 20,000	19(09.55)	31(13.90)	Ref.		Ref.	
20,000-35,000	73(36.68)	58(26.01)	2.05(1.05 to 4.00)	0.03	2.07(0.91 to 4.71)	0.08
35,001-50,000	68(34.17)	82(36.77)	1.35(0.70 to 2.61)	0.37	1.15(0.47 to 2.80)	0.76
> 50,000	39(19.60)	52(23.32)	1.22(0.35 to 1.08)	0.58	1.13(0.42 to 3.05)	0.82
Religion						
Muslim	166(83.42)	192(86.10)	Ref.		Ref.	
Non-Muslim	33(16.58)	31(13.90)	1.23(0.72 to 2.10)	0.44	1.14(0.63 to 2.07)	0.67
Attitude						
Negative	76(38.19)	120(53.81)	Ref.		Ref.	
Positive	123(61.81)	103(46.19)	1.89(1.28 to 2.78)	0.001	1.79(1.16 to 2.76)	0.01
Understanding level						
Poor	73(36.68)	113(50.67)	Ref.		Ref.	
Good	126(63.32)	110(49.33)	1.77(1.20 to 2.62)	0.004	1.74(1.14 to 2.66)	0.01

## 4. Discussion

**Fig 4:**
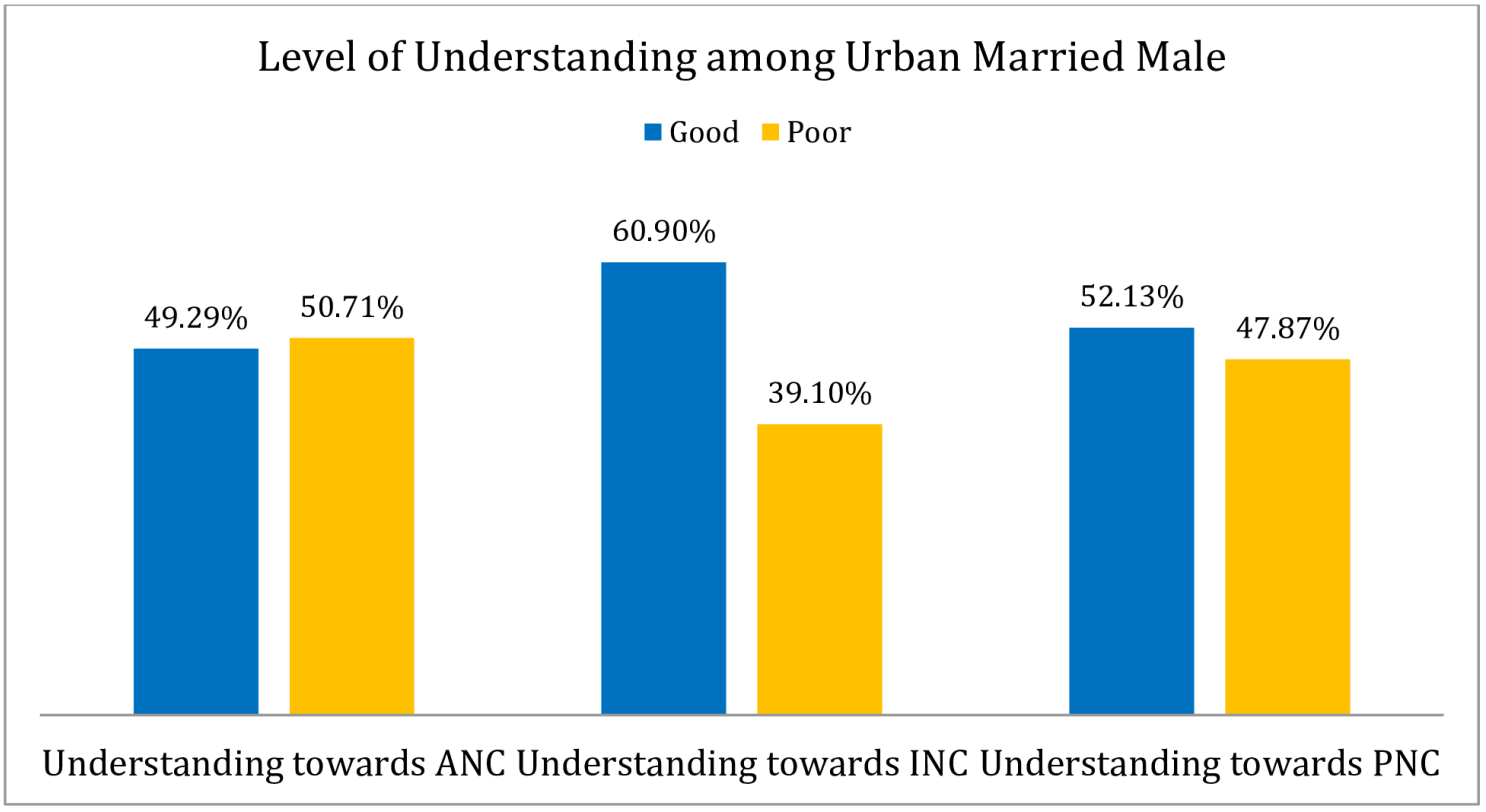
Male partners’ level of understanding towards antenatal, intranatal & postnatal care. Most of the study participants (60.90%) had a good understanding of intranatal care compared to antenatal care (49.29%) as well as postnatal care (52.13%). However, the more frequent poor understanding was related to antenatal care (50.71%) than others care.

**Fig 5:**
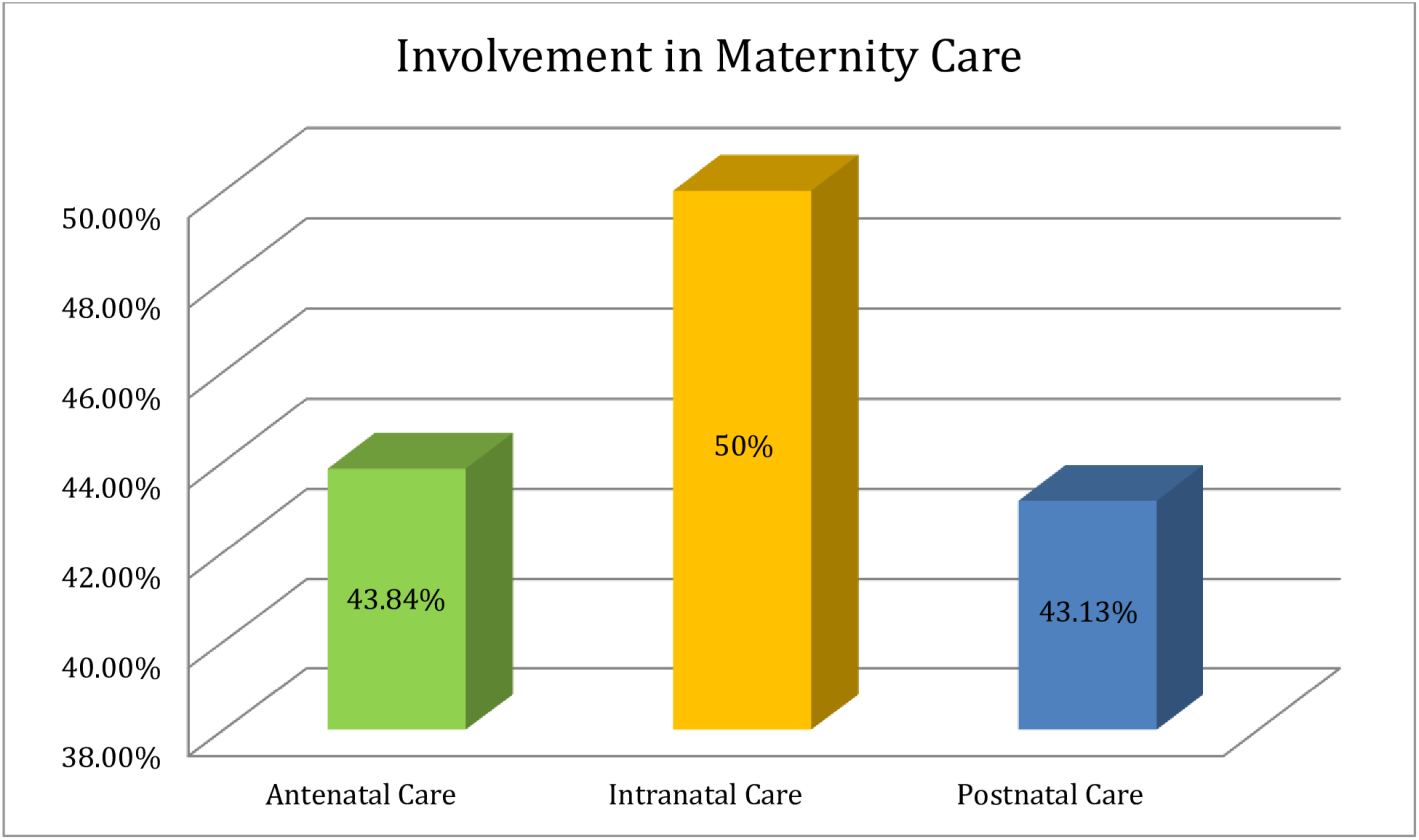
Urban married male partners’ distribution of involvement in maternity care. Approximately 50% of married men joined their spouses during intranatal care, which is more frequent than antenatal (43.84%) and postnatal care (43.13%).

This cross-sectional study primarily examined the understanding, involvement, and associated factors of married male toward maternity care, including antenatal, intranatal, and postnatal care, in an urban setting in Bangladesh. About 55.92% (Fig 1) of the male participants had a good understanding of maternity care, whereas most (60.90%) had a good understanding of intranatal care (Fig 4). A previous study found that the majority of males (63.4%) had knowledge or comprehension of maternity care that was well-informed [10] which is comparatively higher than Bangladesh. On the contrary, a prior investigation in Nigeria showed that 51.5% of respondents had poor understanding of maternal health care [23], which is comparatively higher than this study’s finding (44.08%). This discrepancy might have been visible because of the diverse pedagogy or educational system used in various nations. The bivariate logistic regression model revealed that educational background associated with urban males’ understanding, which is also consistent with this study [10] where it highlighted that knowledge was associated with educational status in respect of maternity care. The occupation of urban married males did not have any link or association with their level of understanding. However, employment status was associated with knowledge regarding maternity care [10]. This inconsistent result could be attributed to the variation in time and geography, and socio-economic structures. Regarding marital status, family size, and the number of children, the logistic regression model could not elicit any significant association with level of understanding of urban married male. An extensive investigation should be conducted taking these factors into account in order to further validate the findings. Furthermore, no association between monthly family income and understanding could be drawn using the bivariate or multivariate logistic regression models. There was no noticeable association of the attitudes of urban married male on their level of understanding; so additional research is necessary to validate the results by taking the association study into consideration. The urban married male partners’ good involvement status in maternity care significantly associated with their knowledge or understanding. A thorough understanding of antenatal, intranatal, and postnatal care can be developed by active participation in maternity care, as explained by the bivariate and multivariate logistic regression models. A previous study found that male partners had a positive attitude toward maternity care but were insufficiently involved due to socio-cultural factors [10]. Approximately 47% of urban married male had good involvement in maternity care, but a previous study [21] conducted in Ibadan, Nigeria, found that overall, 56.9% had good involvement in pregnancy-related care. The observed scenario in this investigation could have been attributed to societal stigma, which was also highlighted in the previous study [21]; where respondents shared that concurrent job demand (68.7%), social stigma (51.8%), and longer waiting times at health facilities (50.7%) as the causes of men’s lack of involvement in pregnancy-related care. It is seen that most of the married male, e.g., about 50%, accompanied their wives during intranatal care higher than antenatal and postnatal care (Fig 5). A previous study revealed that their husbands accompanied approximately 48% 50%, and 63% of women during antenatal, intranatal and postnatal periods, respectively [1]. The urban married males’ age structure had a significant association with their involvement in maternity care, e.g., participants age 42 or older had a lower likelihood of being involved than participants in any other age category, which contradicts the conclusion of earlier studies that there was no significant relation between age category and involvement status [10,16] that might be inconsistence due to socio-demographic diversities. There was no association between educational background and involvement status highlighted in this study through applying bivariate logistic regression model, which is consistent with the following studies [10,24], but the multivariate logistic regression model revealed that there was a link between education and involvement status. It was highlighted by our study finding that males with government jobs were more likely to get involved in maternity care than any other profession, which was strongly supported by a multivariate logistic regression model. However, a prior study showed that having employment decreased the chances of men’s involvement in antenatal care [24]. It can be a result of the employers’ varying self-perceived mindsets. Regarding marital status (family arranged marriage or love marriage), there was no significant relationship with the urban married male’s involvement in maternity care. The degree of engagement varied slightly between forced and planned marriages, as well as between monogamous and polygamous marriages; however, these differences were not statistically significant [24]. Furthermore, there was no discernible association between the involvement of the urban married male partner and family size (nuclear or combined). Further research should be carried out based on cultural and geographical context to validate this conclusion because there are very few studies to compare the current finding. Regarding the number of children, we did not find any significant relationship with urban married males’ involvement in maternity care, which remains constant in the following study [24]. The bivariate logistic regression found that males with family income ranges between 20,000 BDT to 35,000 BDT were more likely to get involved in maternity care, but the multivariate logistic regression model did not support the outcome. A prior study in Bangladesh showed that monthly family income had a significant relationship with male involvement in reproductive health [13]. According to our finding, religious status (Muslim or non-Muslim) not linked to urban married males’ involvement in maternity care. The similar result also found in an earlier investigation [10]. However, another study conducted in Tanzania showed that non-Muslim (Christians) were more likely to be involved in antenatal care than Muslims [24]; the differences might appear due to person specific religious aspects. In addition, about 54% of urban males had a positive attitude towards maternity care, and a prior Nigerian study also showed that about 80.4% respondents had a positive attitude towards maternity care [10], which is higher than this study’s findings. This study highlighted that males who had a positive attitude towards maternity care were more likely to get involved in maternity care, e.g., they were 1.79 times more active in engaging themselves compared to a person who had negative attitudes which did not appear in the previous study conducted in Addis Ababa, Ethiopia [22]. This discrepancy could be emphasized by the fact that attitudes differ among cultures. Gender-specific cultural norms about behavior connected to pregnancy are impeding the adoption of couple-friendly maternity health care services [26]. It was also seen that males with good understanding level were more likely to accompany their wives in maternity care and supported by both the bivariate and multivariate logistic regression model, whereas males with good understanding were 1.74 times more advanced to accompany their wives in multivariate logistic regression model. The previous studies also revealed that a significantly higher proportion of respondents with good knowledge of pregnancy-related care involved in accompanying their partners for antenatal care visits [21], and in Bangladesh, antenatal care visits might be a useful tactic to boost institutional deliveries, which could lower maternal mortality [25], as well as husbands’ knowledge of reproductive health was a significant predictor of male involvement [13]. However, in order to reduce the rate of maternal and infant mortality in a developing country like Bangladesh, the understanding and involvement of urban males in maternity care, as well as the associated factors, must be handled seriously without negligence.

### 4.1 Limitations of the study

There were certain limitations to this study. Firstly, the study’s self-reported data was prone to reporting bias. Therefore, in the next investigation, data triangulation will be quite effective in decreasing reporting bias. Secondly, the convenience sampling technique was confined by selection bias, yet it was somewhat challenging to use alternative probability sampling techniques due to short period of time, resource constraints, and access barriers. Thirdly, other aspects should be considered, such as ethnicity, marriage type (monogamous or polygamous), the frequency of times to visit for antenatal care, and the places of delivery. Fourthly, since this study was conducted in the Dhaka metropolitan area, its findings should not be extrapolated to a rural area. So large scale nationwide survey will be needed to generalize it. Fifthly, most of the males had quite middle to high socioeconomic positions; as a result, it might be necessary to look into people from lower socioeconomic classes. Sixthly, there was a possibility of recall bias in the study as the study participants chosen for the study were between 15–69 years of age, and male above 42 years of age had less involvement in maternal care, this may be due to possible recall bias. Seventhly, birth preparedness classes context in Bangladesh and how is the participation of pregnant women and their husbands in such classes or sessions was not investigated that should be explored in future research through qualitative approach. Finally, the study’s cross-sectional design made it challenging to investigate causality. So, longitudinal study or large-scale research using a mixed-method design should be conducted to further explore these issues.

## 5. Conclusion

Male partners’ appropriate knowledge and participation in maternity care is one of the crucial factors for improving women’s reproductive health. However, the majority of married men in urban areas expressed positive views and revealed a good understanding of maternity care; yet their involvement status was lower than expected. The participation and understanding of urban males, as well as other related aspects linked to maternity care, should be adequately considered to promote safe motherhood activities without inadvertence attitude.

## Supporting information

S1 File Dataset_1. (XLS)

## List of abbreviations

MCH: Maternal and Child Health
ANC: Antenatal Care
INC: Intranatal Care
PNC: Postnatal Care
WHO: World Health Organization
HIV: Human Immunodeficiency Virus
MDG: Millennium Development Goal
SDG: Sustainable Development Goal
MMR: Maternal Mortality Ratio
IMR: Infant Mortality Rate
BDT: Bangladesh Taka
USD: United States Dollar
SSC: Secondary School Certificate
HSC: Higher Secondary Certificate
